# Synergistic Effect of *Bacillus thuringiensis* IAGS 199 and Putrescine on Alleviating Cadmium-Induced Phytotoxicity in *Capsicum annum*

**DOI:** 10.3390/plants9111512

**Published:** 2020-11-08

**Authors:** Anis Ali Shah, Fatima Bibi, Iqtidar Hussain, Nasim Ahmad Yasin, Waheed Akram, Muhammad Saeed Tahir, Hayssam M. Ali, Mohamed Z. M. Salem, Manzer H. Siddiqui, Subhan Danish, Shah Fahad, Rahul Datta

**Affiliations:** 1 Department of Botany, University of Narowal, Narowal 51801, Pakistan; anisalibot@gmail.com (A.A.S.); fatimabibibot@gmail.com (F.B.); 2 Department of Agronomy, Faculty of Agriculture, Gomal University, Dera Ismail Khan 29050, Pakistan; iqtidarhussain453@yahoo.com; 3 Senior Suprintendent Gardens, Resident Officer-II office Department, University of the Punjab, Lahore 54590, Pakistan; 4 Vegetable research institute, Guangdong Academy of Agriculture Science, Guangzhou 510640, China; meher_waheed@yahoo.com; 5 Department of Agronomy, Faculty of Agricultural Sciences and Technology, Bahauddin Zakariya University, Multan 60800, Pakistan; saeedtahir57@yahoo.com; 6 Department of Botany and Microbiology, College of Science, King Saud University, Riyadh 2455, Saudi Arabia; hayhassan@ksu.edu.sa (H.M.A.); mhsiddiqui@ksu.edu.sa (M.H.S.); 7 Timber Trees Research Department, Sabahia Horticulture Research Station, Horticulture Research Institute, Agriculture Research Center, Alexandria 21526, Egypt; 8 Forestry and Wood Technology Department, Faculty of Agriculture (El-Shatby), Alexandria University, Alexandria 21545, Egypt; zidan_forest@yahoo.com; 9 Department of Soil Science, Faculty of Agricultural Sciences and Technology, Bahauddin Zakariya University, Multan 60800, Pakistan; 10 Hainan Key Laboratory for Sustainable Utilization of Tropical Bioresource, College of Tropical Crops, Hainan University, Haikou 570228, China; 11 Department of Agronomy, The University of Haripur, Haripur 22620, Pakistan; 12 Department of Geology and Pedology, Faculty of Forestry and Wood Technology, Mendel University in Brno, Zemedelska 3, 61300 Brno, Czech Republic

**Keywords:** cadmium, *Capsicum annum*, growth, microbe, priming, putrescine

## Abstract

Plant growth-promoting bacteria (PGPB) and putrescine (Put) have shown a promising role in the mitigation of abiotic stresses in plants. The present study was anticipated to elucidate the potential of *Bacillus thuringiensis* IAGS 199 and Put in mitigation of cadmium (Cd)-induced toxicity in *Capsicum annum*. Cadmium toxicity decreased growth, photosynthetic rate, gas exchange attributes and activity of antioxidant enzymes in *C. annum* seedlings. Moreover, higher levels of protein and non-protein bound thiols besides increased Cd contents were also observed in Cd-stressed plants. *B. thuringiensis* IAGS 199 and Put, alone or in combination, reduced electrolyte leakage (EL), hydrogen peroxide (H_2_O_2_) and malondialdehyde (MDA) level in treated plants. Synergistic effect of *B. thuringiensis* IAGS 199 and Put significantly enhanced the activity of stress-responsive enzymes including peroxidase (POD), ascorbate peroxidase (APX), catalase (CAT) and superoxide dismutase (SOD). Furthermore, Put and microbial interaction enhanced the amount of proline, soluble sugars, and total soluble proteins in *C. annum* plants grown in Cd-contaminated soil. Data obtained during the current study advocates that application of *B. thuringiensis* IAGS 199 and Put establish a synergistic role in the mitigation of Cd-induced stress through modulating physiochemical features of *C. annum* plants.

## 1. Introduction

Cadmium is one of the environmental toxicants that hampers the growth of numerous crops. Cadmium is present in color pigments, PVC products and Ni-Cd batteries. It is released into the environment as a result of smelting, fossil fuel combustion and use of phosphate fertilizers [1]. Cadmium interferes with the activity of antioxidant enzymes, thereby reduces the normal physiochemical activities in plants. [2,3]. This metal contaminant has been destroying cultivated areas and is limiting crop choice in polluted areas [4]. The increasing Cd contents in soil owing to anthropogenic and natural resources have a detrimental effect on physiochemical attributes causing reduced crop growth and biomass production [5,6]. Biochemical such as C–O bonds, protein-containing thiol groups, amine groups and carbonyl are involved in Cd uptake and accumulation in plants [7]. Uptake and translocation of Cd to comestible plant parts is a widespread source of Cd exposure for living organisms, ensuing substantial health risks [8]. Cadmium also decreases the concentration of essential mineral nutrients by declining their uptake and translocation as it obstructs the entry of these nutrients [9]. It also decreases thye production of chlorophyll in plants [10,11,12]. This toxic metal demonstrates a resilient affinity with sulfhydryl moiety of enzymes and enhances the biosynthesis of reactive oxygen species (ROS) leading to oxidative stress [13]. Oxidative stress reduces the turgidity of nucleic acids, proteins and cell membranes [14]. Alternatively, the inhibition or over-expression of genes related to metal transportation and chelation including metallothioneins (MTs) and phytochelatins synthase (PCS), supports plants to mitigate metal stress [15]. Stress relevant enzymes, antioxidants and other osmoprotectants also assist plants to reduce the extent of oxidative injuries through maintenance of cellular water contents [14]. 

Polyamines (PAs) for instance Put, spermine (Spm) and spermidine (Spd) are minute polycations. These light weighted molecules are present in tissues of a number of plants and microbial species. These biomolecules regulate physiological and metabolic activities enabling plants to alleviate different kinds of environmental stresses [16]. PAs join with cellular polymers and molecules to be utilized as osmosis regulating solutes. Ammonia propyl is donated by methionine ensuing synthesis of Putrescine (Put), leading to the biosynthesis of Spm and Spd. Hence, Put is the basic biomolecule present in the synthetic pathway of PAs. It was observed that Put improved antioxidative activity of enzymes and reduced lipid peroxidation level in salt-stressed pine seedlings [17]. Put induces the biosynthesis of other PAs [18]. Consequently, the increased biosynthesis of indigenous PAs enables plants to scavenge ROS and alleviate various kinds of environmental stresses [16]. The ability of Put to stabilize plant cell walls and neutralize acid enables it to mitigate environmental stress [19]. The exogenous application of Put also improves enzymatic activity, rate of photosynthesis, seed germination, seedling vigor and overall growth of plants facing environmental stress [13]. Rhizobacteria living in the roots zone of plants and improve the growth of these plants either directly or indirectly are termed as plant growth-promoting bacteria (PGPB) [10,20,21,22,23,24,25,26,27]. Soil rhizobacteria play a significant role in phytoremediation of contaminated soil, increase nutrient uptake and promote plant growth and yield [25,27,28,29,30]. PGPB are capable of maintaining growth-promoting attributes of plants via communal protein stimulation by PGPB–plant communications during and post-colonization [31]. Seed priming with PGPB inoculum is termed as biopriming. Bioprimed seeds allow adhered PGPB to colonize and enhance germination speed resulting in the rapid establishment of crop plants and subsequently higher yield [32]. These PGPB are capable to increase plant growth and alleviate abiotic stress through secretion of phytohormones like IAA and by modulating physiochemical attributes of assisted plants [21,22,23,27,33]. Naser et al. [34] informed that PGPB inoculation mitigated salt-induced oxidative stress in *Phoenix dactylifera*. Similarly, Etesami [35] reported that PGPB reduce metal uptake, phytotoxicity and enhance growth and biomass production in inoculated plants. Furthermore, the PGPB capable of inducing metal precipitation, bioaccumulation, chelation and complexation diminish phytotoxic effects in supplemented plants [36]. 

Keeping this in view, it was hypothesized that seed priming and subsequent application of *B. thuringiensis* IAGS 199 may improve the growth of *C. annum* plants exposed to Cd stress. The research was designed to evaluate the potential of *B. thuringiensis* IAGS 199, alone or in combination with Put, in the alleviation of Cd stress in *C. annum* seedlings. Furthermore, the effect of Put and *B. thuringiensis* IAGS 199 on the synthesis of metal-chelating biochemicals such as protein and non-protein bound thiols and total thiols were also evaluated. The use of biostimulants reduces the need for fertilizers [37,38,39]. Foliar application of fertilizer and micronutrient could be a better alternative to fast action [40,41].

## 2. Results

### 2.1. Cadmium Immobilization and Putrescine Concentration in Solution

**Figure 1:**
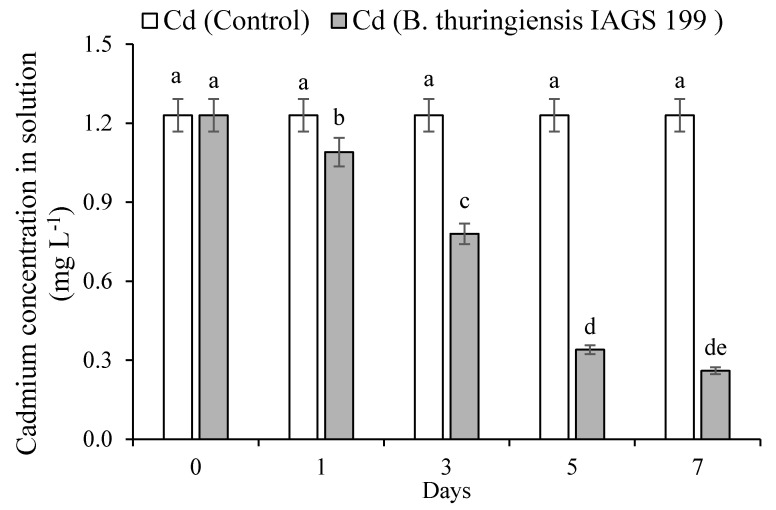
Changes in the cadmium concentration in the culture solution in the presence of *B. thuringiensis* IAGS 199. Means are an average of five replicates. Different letters on bars showed a statistical difference at *p* < 0.05.

**Figure 2:**
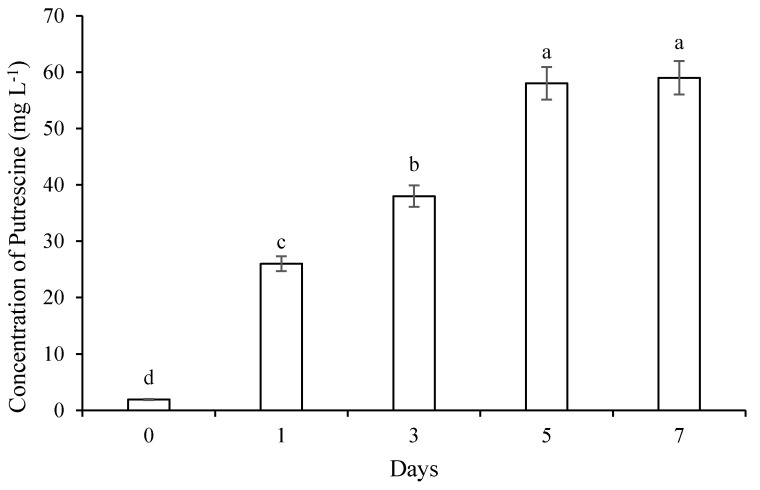
Changes in the Put concentration in the culture solution in the presence of *B. thuringiensis* IAGS 199. Means are an average of five replicates. Different letters on bars showed a statistical difference at *p* < 0.05.

As shown in Figure 1, water-soluble Cd concentration did not differ significantly in the absentia of *B. thuringiensis* IAGS 199 strain. However, water-soluble Cd concentration reduced significantly in the presence of a microbe. The pH ranged from 6.24 to 6.85 in the presence as well as absentia of the strain. Figure 2 explains that Put concentration elevated in the presence of a microbe. *B. thuringiensis* IAGS 199 strain produced Put. After 7 days, the peak value of Put was 59 mg L^−1^. Moreover, Cd content on the cell surface of *B. thuringiensis* IAGS 199 strain increased with time, ranging from 0.03 mg g^−1^ to 0.09 mg g^−1^ between 1 and 7 days of incubation. Intracellular Cd content also varied from 0.10–0.17 mg g^−1^.

### 2.2. Analysis of Growth Parameters

The results showed that Cd toxicity significantly reduced germination percentage, root fresh weight, shoot fresh weight, root dry weight and shoot dry weight by 43.85%, 86.56%, 58.73%, 40.8% and 55.47%, respectively, as compared to control treatment. Application of *B. thuringiensis* IAGS 199 and Put enhanced growth attributes in normal and Cd-contaminated soil. In case of *C. annum* seedlings grown in Cd-contaminated soil, the combined application of *B. thuringiensis* IAGS 199 and Put significantly enhanced growth attributes of *C. annum* seedlings as compared to individual treatment of *B. thuringiensis* IAGS 199 and Put (Table 1).

### 2.3. Quantification of Chlorophyll Contents

Cadmium stress reduced Chl content in *C. annum* seedlings as compared to control. Individual treatments of *B. thuringiensis* IAGS 199 and Put enhanced Chl a, Chl b and total chlorophyll content in *C. annum* seedlings. Nevertheless, co-application of *B. thuringiensis* IAGS 199 and Put significantly enhanced Chl contents in *C. annum* seedlings under Cd contaminated conditions (Table 2). 

### 2.4. Determination of Photosynthetic Rate and Gas-Exchange Parameters

**Figure 3:**
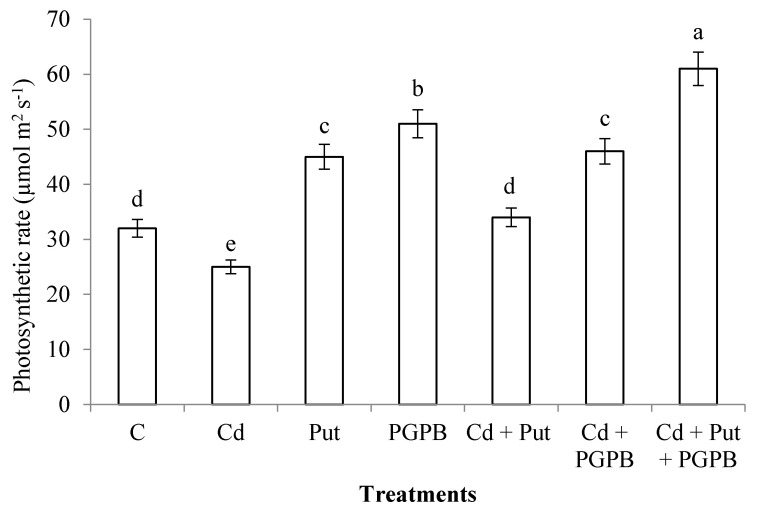
Effect of Put and *B. thuringiensis* IAGS 199 on photosynthetic rate of *C. annum* under Cd stress. Values demonstrate means ± SD (*n* = 5). Different letters indicate significant difference among the treatments (*p* ≤ 0.05). C, control; Cd, 50 mg kg^−1^ Cd; Put, 1 mM Put; PGPB, *B. thuringiensis* IAGS 199.

Cadmium stress decreased photosynthetic rate in *C. annum* plants by 28%, as compared to control. Application of *B. thuringiensis* IAGS 199 and Put enhanced photosynthetic rate in normal and Cd-polluted soil, as compared with control and Cd-treatment, respectively. However, the synergistic application of *B. thuringiensis* IAGS 199 and Put significantly enhanced the photosynthetic rate of *C. annum* plants, as compared to alone treatments of *B. thuringiensis* IAGS 199 and Put (Figure 3). 

**Figure 4:**
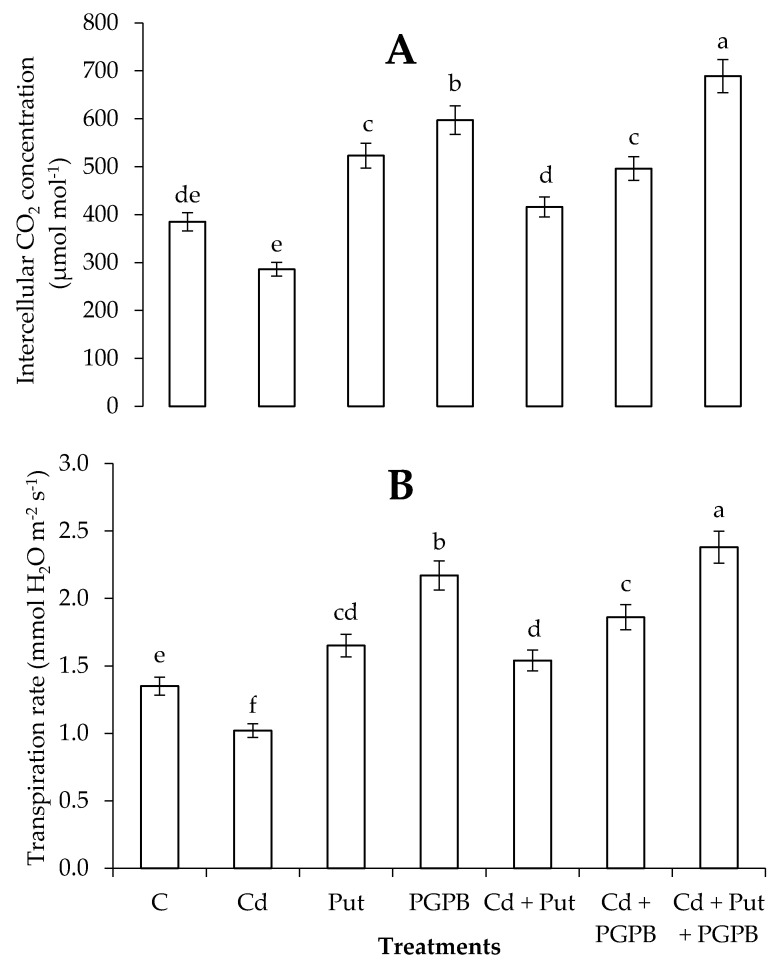
Effect of Put and *B. thuringiensis* IAGS 199 on Intercellular CO_2_ concentration (**A**) and Transpiration rate (**B**) of *C. annum* under Cd stress. Values demonstrate means ± SD (*n* = 5). Different letters indicate significant difference among the treatments (*p* ≤ 0.05). C, control; Cd, 50 mg kg^−1^ Cd; Put, 1 mM Put; PGPB, *B. thuringiensis* IAGS 199. Both A and B have same x-axix.

Cadmium stress decreased gas exchange attributes of *C. annum* plants, as compared to control treatment. Combined application of *B. thuringiensis* IAGS 199 and Put significantly enhanced intercellular CO_2_ concentration and transpiration rate in *C. annum* plants, in assessment with alone treatments of *B. thuringiensis* IAGS 199 and Put in normal and Cd-toxic soil (Figure 4). 

### 2.5. Analysis of Lipid Peroxidation and Hydrogen Peroxide Content

**Figure 5:**
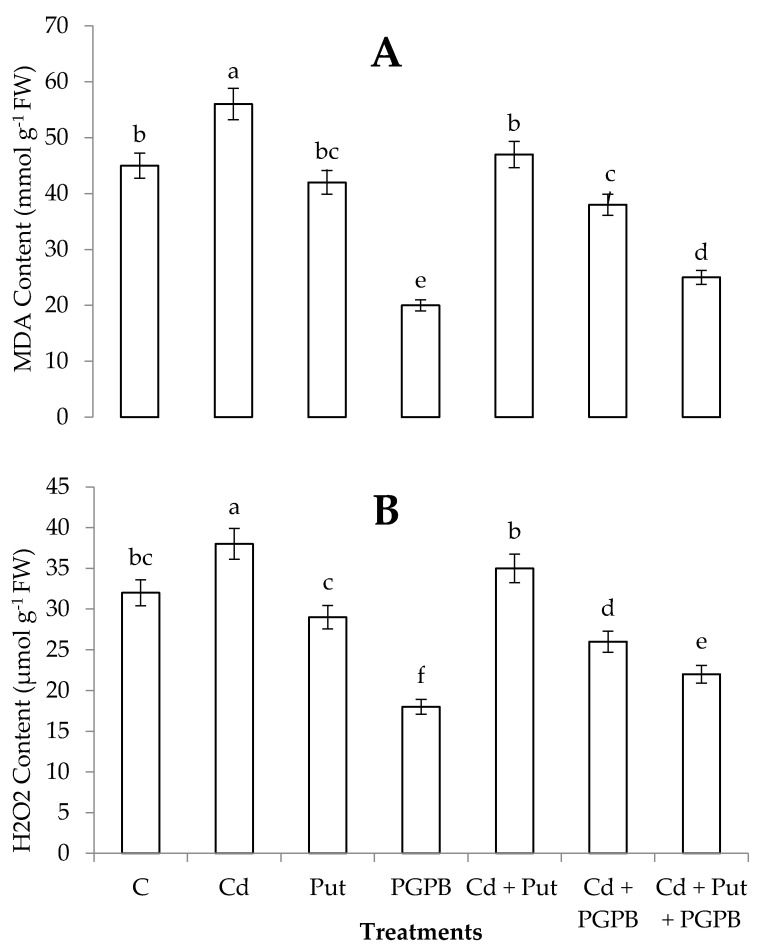
Effect of Put and *B. thuringiensis* IAGS 199 on malondialdehyde (MDA) (**A**) and hydrogen peroxide (H_2_O_2_) (**B**) contents of *C. annum* under Cd stress. Values demonstrate means ± SD (*n* = 5). Different letters indicate significant difference among the treatments (*p* ≤ 0.05). C, control; Cd, 50 mg kg^−1^ Cd; Put, 1 mM Put; PGPB, *B. thuringiensis* IAGS 199. Both A and B have same x-axix.

Cadmium stress significantly enhanced MDA content (24.44%) in *C. annum* plants, in comparison with control treatment. Alone treatment of *B. thuringiensis* IAGS 199 and Put reduced MDA content in *C. annum* plants grown in normal and Cd-contaminated soil. Nevertheless, synergistic treatment of *B. thuringiensis* IAGS 199 and Put reduced MDA content in the case of *C. annum* seedlings grown in Cd-contaminated soil (Figure 5).

Cadmium toxicity augmented H_2_O_2_ contents (18.75%) in *C. annum* plants, as compared to control treatment. In the case of *C. annum* seedlings grown in non-contaminated conditions, alone treatments of Put and *B. thuringiensis* IAGS 199 reduced H_2_O_2_ contents significantly as compared to Cd-treatment. In Cd-contaminated soil, synergistic application of Put and *B. thuringiensis* IAGS 199 significantly reduced H_2_O_2_ contents in comparison with alone treatments of Put and *B. thuringiensis* IAGS 199 (Figure 5). 

### 2.6. Determination of Total Soluble Protein and Soluble Sugars

Cadmium stress decreased total soluble proteins by 55.55%, as compared to control treatment. Individual treatments of Put and *B. thuringiensis* IAGS 199 elevated total soluble proteins content in *C. annum* seedlings grown in normal and Cd-polluted soil. Nevertheless, the co-application of Put and *B. thuringiensis* IAGS 199 significantly enhanced total soluble proteins under Cd-stress. Conversely, Cd stress enhanced the soluble sugar level in *C. annum* seedlings. Individual treatments of Put and *B. thuringiensis* IAGS 199 enhanced soluble sugar content in normal and Cd-polluted soil. The highest soluble sugar content was found when treated with *B. thuringiensis* IAGS 199 in normal soil (Table 2).

### 2.7. Determination of Electrolyte Leakage (EL)

**Figure 6:**
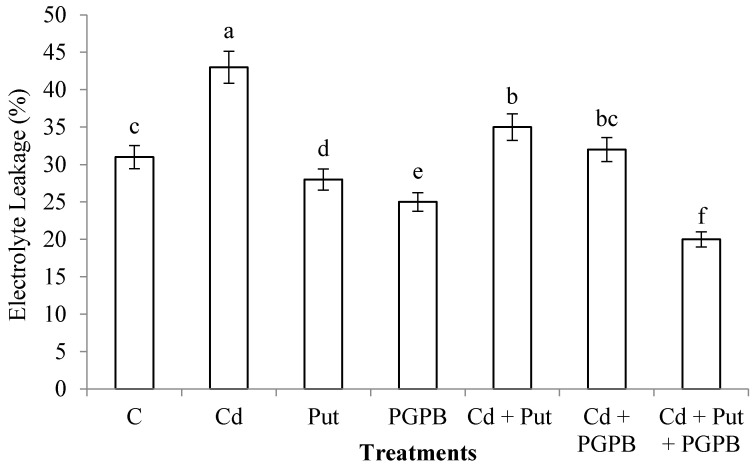
Effect of Put and *B. thuringiensis* IAGS 199 on EL of *C. annum* under Cd stress. Values demonstrate means ± SD (*n* = 5). Different letters indicate significant difference among the treatments (*p* ≤ 0.05). C, control; Cd, 50 mg kg^−1^ Cd; Put, 1 mM Put; PGPB, *B. thuringiensis* IAGS 199.

Cadmium stress significantly enhanced EL (38.70%) as compared to control treatment. *B. thuringiensis* IAGS 199 and Put reduced EL in *C. annum* plants grown in normal and Cd-toxic soil. Nevertheless, synergistic application of *B. thuringiensis* IAGS 199 and Put significantly reduced EL in *C. annum* plants grown in Cd-toxic soil (Figure 6). 

### 2.8. Evaluation of Antioxidant Enzymatic Activities

**Figure 7:**
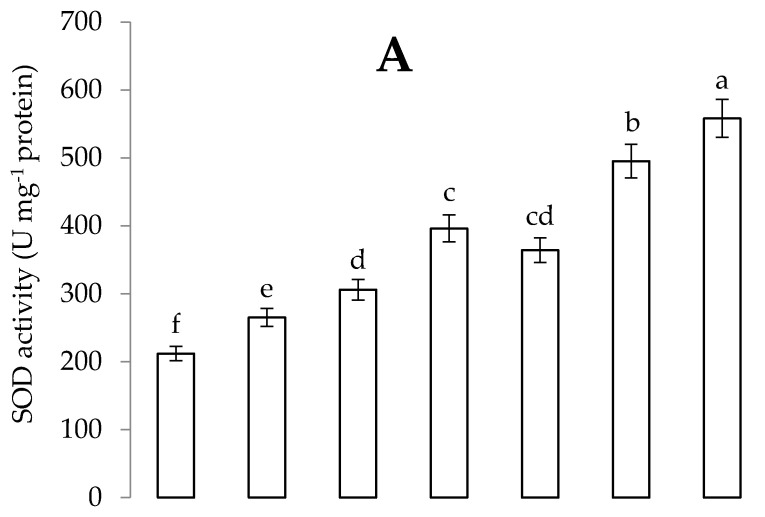

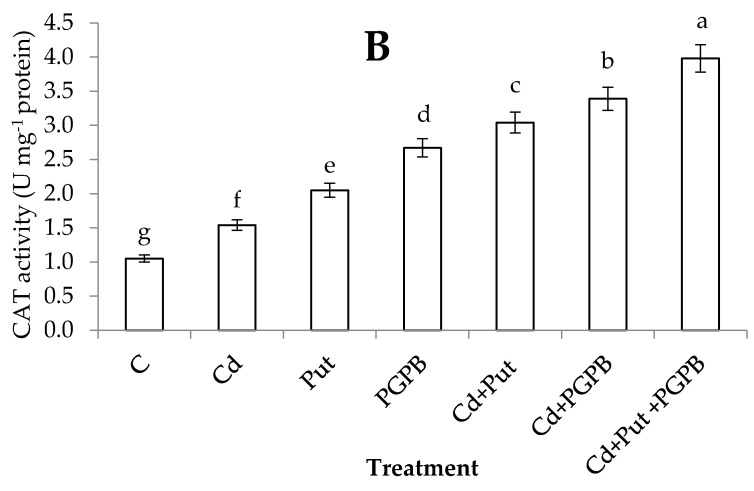
Effect of Put and *B. thuringiensis* IAGS 199 on superoxide dismutase (SOD) (**A**) and catalase (CAT) (**B**) activity of *C. annum* under Cd stress. Values demonstrate means ± SD (*n* = 5). Different letters indicate significant difference among the treatments (*p* ≤ 0.05). C, control; Cd, 50 mg kg^−1^ Cd; Put, 1 mM Put; PGPB, *B. thuringiensis* IAGS 199. Both A and B have same x-axix.

**Figure 8:**
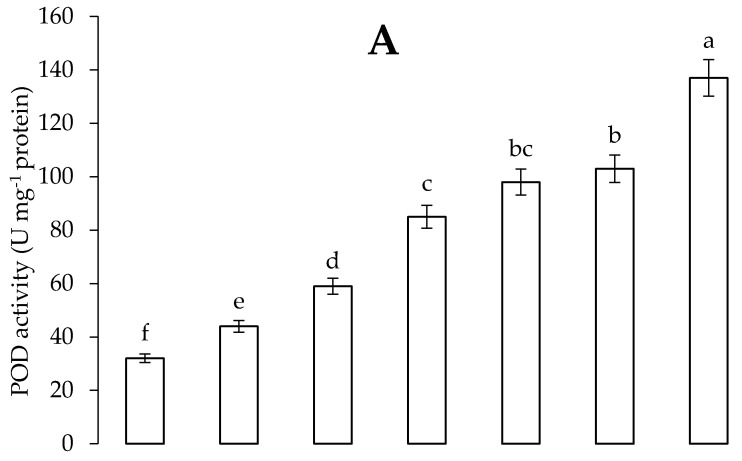

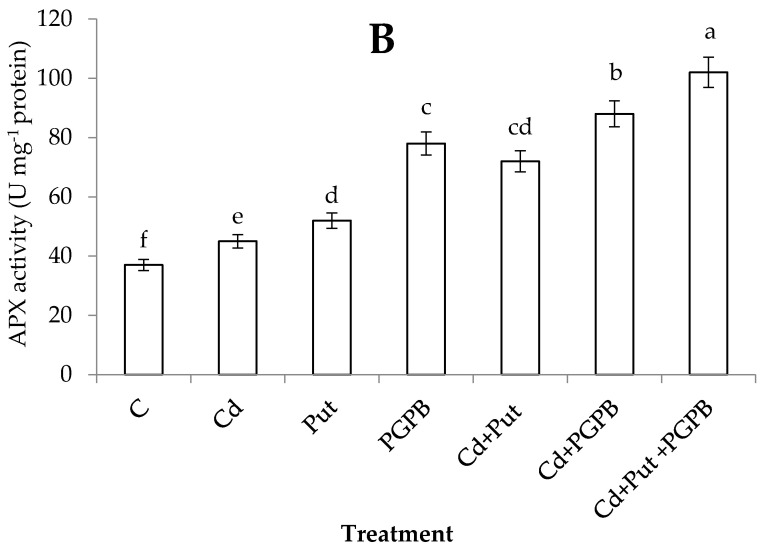
Effect of Put and *B. thuringiensis* IAGS 199 on peroxidase (POD) (**A**) and ascorbate peroxidase (APX) (**B**) activity of *C. annum* under Cd stress. Values demonstrate means ± SD (*n* = 5). Different letters indicate significant difference among the treatments (*p* ≤ 0.05). C, control; Cd, 50 mg kg^−1^ Cd; Put, 1 mM Put; PGPB, *B. thuringiensis* IAGS 199. Both A and B have same x-axix.

Cadmium stress augmented the levels of SOD, CAT, POD and APX enzyme in *C. annum* plants. Application of *B. thuringiensis* IAGS 199 and Put enhanced the activity of antioxidant enzymes in *C. annum* plants. However, synergistic application of *B. thuringiensis* IAGS 199 and Put in Cd-contaminated soil significantly incremented levels of SOD, CAT, POD and APX enzyme in *C. annum* plants (Figure 7 and Figure 8). 

### 2.9. Determination Proline Contents 

**Figure 9:**
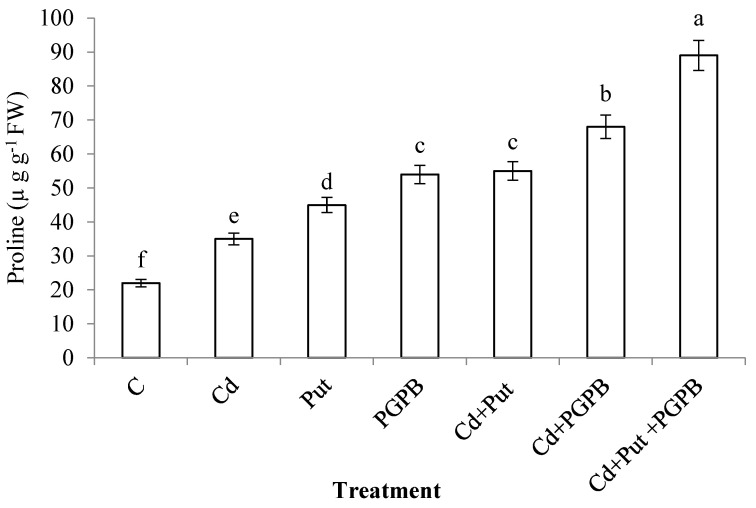
Effect of Put and *B. thuringiensis* IAGS 199 on proline content of *C. annum* under Cd stress. Values demonstrate means ± SD (*n* = 5). Different letters indicate significant difference among the treatments (*p* ≤ 0.05). C, control; Cd, 50 mg kg^−1^ Cd; Put, 1 mM Put; PGPB, *B. thuringiensis* IAGS 199.

Cadmium stress enhanced the activity of proline content (59.09%) in *C. annum* plants as compared to control treatment. In the case of *C. annum* plants grown in Cd-toxic conditions, synergistic application of *B. thuringiensis* IAGS 199 and Put significantly enhanced proline levels in *C. annum* plants as compared to Cd-treatment (Figure 9).

### 2.10. Analysis of Cd Content

Table 3 shows that Cd content decreased in shoots of *C. annum* seedlings, as compared to Cd content in the root. Co-treatment of *B. thuringiensis* IAGS 199 and Put significantly decreased Cd contents in the shoot of *C. annum* seedlings, as compared to alone treatments of *B. thuringiensis* IAGS 199 and Put in Cd-contaminated conditions. In the case of *C. annum* seedlings grown in normal and Cd-contaminated conditions, alone treatment of *B. thuringiensis* IAGS 199 in normal conditions and combined application of *B. thuringiensis* IAGS 199 and Put in Cd-stressed conditions showed significantly higher value of MTI, respectively.

### 2.11. Determination of Metal Chelating Compounds

Cadmium stress enhanced total thiol content (54.16%) in *C. annum* seedlings as compared to control treatment. Alone application of Put and *B. thuringiensis* IAGS 199 significantly enhanced total thiol content in Cd-toxic soil, as compared to *C. annum* seedlings grown in non-contaminated soil. In the case of *C. annum* seedlings grown in Cd-contaminated soil, synergistic application of Put and *B. thuringiensis* IAGS 199 significantly enhanced total thiol content as compared to alone treatments of Put and *B. thuringiensis* IAGS 199. Moreover, non-protein thiol and protein thiol content was also significantly enhanced by the co-application of Put and *B. thuringiensis* IAGS 199 in Cd-contaminated soil, as compared to other treatments (Table 4).

## 3. Discussion

Put is an essential biogenic cationic polyamine in microbes, plants, and animals [42]. Beatriz et al. [43] reported that *Lactococcus lactis* is able to synthesize Put to induce alkalinization of the culture solution. During current research, *B. thuringiensis* IAGS 199 enhanced Put and increased pH yet reduced Cd concentration in the culture solution. It may be assumed that *B. thuringiensis* IAGS 199 immobilized Cd by enhancing Put synthesis and pH in the culture solution (Figure 1). The Cd was adsorbed on the cell surface, intracellular and extracellular regions of *B. thuringiensis* IAGS 199 (Figure 2). *B. thuringiensis* IAGS 199 enhanced Put synthesis causing immobilization of Cd in the solution (Figure 1). Bacteria tolerate metal toxicity through (1) sequestrating metal on their cell walls or by employing metallothioneins and phytochelatins which are intracellular metal-binding proteins and peptides (2) amending uptake paths thus hindering metal uptake; (3) converting toxic form of metal to a less toxic form by enzymatic activities; and (4) reducing the intracellular metal concentration through engaging a particular efflux scheme or through compartmentalization [44]. The PGPB–plant association affects the availability and uptake of Cd in plants through chelation, precipitation, bioaccumulation and complexation [36]. The bacterial strains capable to synthesize siderophores, auxin and ACCD improve growth in metal-stressed *C. annum* plants [45]. Jebara et al. [46] also revealed that PGPB synthesizing phytohormones such as auxin and capable to solubilize P and fix atmospheric N assist companion plants to improve growth and alleviate metal stress. The ACCD activity of PGPB maintains the synthesis of stress ethylene in plants under metal stress [47]. The arginine decarboxylase synthesizing PGPR induce numerous physiochemical changes in metal-stressed plants to alleviate respective stress [48]. *B. thuringiensis* IAGS 199 inoculated *C. annum* plants exhibited improved growth in comparison with the control in the non-contaminated and Cd spiked soils (Figure 2). *B. thuringiensis* IAGS 199 revealed different plant growth-promoting attributes responsible for Cd stress alleviation and growth promotion of treated *C. annum* plants (Figure 4). Bacterial inoculation may induce enhanced Put synthesis, Cd immobilization and reduced Cd contents in crop plants [49,50]. Plants inoculated with *B. thuringiensis* IAGS 199 decreased the amount of soil available Cd (Figure 5), reduced Cd uptake in plants as compared to the control (Figure 4). Moreover, reduced translocation factor of *B. thuringiensis* IAGS 199 applied plants may be ascribed to the declined Cd accumulation in inoculated plants compared to the control (Table 1). *B. thuringiensis* IAGS 199 enhanced the concentration of Put and ADPB in soils, causing increased pH. The high pH results from the insolubilization of Cd by making hydroxides, organic complexes and carbonates precipitates [51]. 

Madhaiyan et al. [52] also reported reduced Ni and Cd bioavailability and uptake in tomato plants treated with *Magnaporthe oryzae* CBMB20 and *Burkholderia* sp. CBMB40, respectively. Similarly, Lin et al. [53] observed that Cd resistant *B. megaterium* H3 decreased Cd bioavailability, uptake and accumulation in rice plants. The metal-resistant PGPR reduce the bioavailability of metal through binding metal on their cell surfaces or accumulating in their cells (Ledin et al. 1999). The Cd precipitation, intracellular and extracellular adsorption accumulation by *B. thuringiensis* IAGS 199 (Figure 2 and Figure 3), caused a decrease in bioavailable Cd in the soil. Metal-resistant PGPR may chelate metal ions through their siderophores and reduce the bioavailability of these metals [54,55]. *B. thuringiensis* IAGS 199 may have synthesized siderophores causing chelation and binding of Cd resulting decreased Cd bioavailability in soil and reduced Cd uptake and bioaccumulation in inoculated plants (Table 1, Figure 5). Furthermore, *B. thuringiensis* IAGS 199 enhanced root growth which ultimately increased synthesis of root exudates and concentration of organic matter in the rhizospheric soil. The higher concentration of organic matter content enhanced ADPB in *B. thuringiensis* IAGS 199 supplemented soils compared to the controls. The increased synthesis of polyamines assists plants to mitigate heavy metal stress [56,57]. The higher concentration of organic matter besides increased ADPB and Put in *B. thuringiensis* IAGS 199 inoculated rhizospheric soils may be responsible for improving the growth of *C. annum* plants and the immobilization of Cd in the Cd amended soil.

Polyamines (PAs) assist plants to alleviate stresses through modulation of physiochemical activities and reduction of toxic metals uptake [58]. Our current study reveals the role of Put and *B. thuringiensis* IAGS 199 in the alleviation of Cd-induced phytotoxicity in *C. annum*. The uptake and accretion of Cd impede cellular growth besides the activity of proton pumps causing variations in the growth configurations and physiological activities [59]. Nevertheless, Put-treated *C. annum* seedlings were capable to ameliorate Cd-induced stress and exhibited improved growth. Put application triggered an improvement in seed germination, root length, shoot length and biomass production in alfalfa [60]. Zhao et al. [61] demonstrated that γ-Aminobutyric acid (GABA) regulates lipid production and Cd uptake in plants. Since GABA induces synthesis of PAs. Therefore, it is assumed that increased biosynthesis of Put in response to GABA alleviated Cd-induced toxicity in *C. annum* plants through the reduction of lipid peroxidation. Some other researchers have also supported the metal stress mitigation capability of PAs in different plants [62].

Li et al. [6] reported that *B. thuringiensis* HC-2 reduced Cd content in radish roots. The results of our experiments also showed that *B. thuringiensis* IAGS 199, alone or in combination with Put, reduced Cd uptake in shoots of *C. annum* seedlings. 

Equally, the reduced levels of protein contents were observed in Cd supplemented plants (Table 2), while an improved amount of amino acids and proline also appears to be a tactic of seed plants to alleviate Cd toxicity through scavenging a higher amount of ROS by employing osmoregulators. Put residues are a source of spermidine and spermine biosynthesis [63]. Spermine mitigates abiotic plant stress through modulation of glyoxalase system and antioxidative machinery [64]. Likewise, spermidine alleviates Cd phytotoxicity through intonation of nitrogen metabolism and antioxidative machinery [14]. It was detected that Put treatment enhanced photosynthetic pigments, growth parameters, soluble protein contents and proline contents in *C. annum* seedlings under Cd regimes. 

The reduced level of electrolyte leakage and Cd content was perceived in Put applied *C. annum* seedlings. The improved level of growth attributes in Put-treated Cd-stressed seedlings may be a result of reduced uptake and translocation of Cd in plant tissues (Table 3). Potassium acts as an osmolyte and helps in the maintenance of membranous stability. Put maintains the dimensions of K^+^ channel and porosity in guard cells and regulates transpiration in plants [65]. Catabolites of Put regularize uptake and translocation of Ca^2+^ and K^+^, and therefore maintain bioenergetics of chloroplast and mitochondria under stress [66]. Rahdari et al. [67] also found that Put regularizes the biosynthesis of photosynthetic pigments in plants subjected to abiotic stresses. Higher K^+^ ions help in the maintenance of membrane stability and reduce the level of electrolyte leakage in stressed seedlings [68]. By the same token, higher uptake of macronutrient cations like N, P and K perhaps restricted Cd uptake [69]. Higher K^+^ ions altered the biosynthesis level of endogenous hormones and in return enhanced chlorophyll fluorescence [70] in Put-treated seedlings. Polyamines maintained water integrity, reduced Cd content in Put-treated seedlings confer beneficial role of this PA for LRWC. Improved membranous integrity and stress alleviation in PAs applied plants was reported by Groppa et al. [57]. Cadmium phytotoxicity adversely affected water relations and biomass production of subjected *C. annum* plants (Table 1). Metal toxicity reduces water uptake, transpiration, growth and biomass production in the stressed plant was reported by Rady et al. [71].

*B. thuriengiensis* is involved in the enhancement of Ca^++^, Mg^++^, Zn^++^, Mn^++^, and Cu^++^, and enhancement of nutritional content assisted in drought stress management in *Lavandula angustifolia* and *Salvia divinorum* [72]. Calcium is involved in membrane stabilization and Mg^++^ accumulation in chloroplasts [73]. Current research also depicts the involvement of *B. thuringiensis* IAGS 199, alone or in combination with Put, in the enhancement of photosynthetic rate and pigment content in *C. annum* seedlings.

Li et al. [74] found that Put application assisted in membranous stability and standardization of the active oxygen biosynthesis; by this means, Put application defended plants from acid toxicity and improved their stress tolerance. A higher amount of EL was observed in Cd-stressed plants (Figure 4), confirming the involvement of Cd-induced phytotoxicity in the stability and permeability of the cellular membrane. Hassan et al. [75] demonstrated that exogenously applied Put alleviate plant stress through the protection of chloroplast and membranous structure. Our study also showed that Pu application, alone or in combination with *B. thuringiensis* IAGS 199, protected *C. annum* seedlings from Cd toxicity by stabilization of photosynthetic apparatus. 

A higher level of osmoregulators including proline and soluble sugars and reduced level of protein content was observed in Cd-stressed *C. annum* seedlings. Put treatment further enhanced proline contents in Cd-stressed plants. Higher proline contents in Put applied plants confer linkage between PAs metabolism to proline biosynthesis [76,77]. Our results are analogous to Sharma and Dietz [78] who reported protein degradation resulting in an upgraded level of amino acids in stressed plants. Higher biosynthesis of proline helps in stress mitigation through chelation and metal detoxification, ROS scavenging, osmoregulation, enzymatic defense, and modulation of cytosolic acidity. Sun et al. [79] revealed that exogenous Put application declined MDA content while the enhanced amount of photosynthetic pigments, proline content and activity of antioxidant enzymes in *Anthurium andraeanum* under chilling stress. The increased synthesis of osmoregulators including soluble sugars, proline and free amino acids helps to alleviate drought stress and improve the growth of Put-treated plants [80]. In the same way, the increased amount of free amino acids and proline in Put applied seedlings assisted in the reduction of Cd-induced toxicity. Further enhancement of total free amino acids in Put-treated seedlings enabled plants to manage Cd stress in a better way.

*B. subtilis* improved Cd tolerance in *Triticum aestivum* through enhancing the activity of POD, CAT besides reducing the level of MDA [81]. In another study, Jan et al. [82] reported that *B. cereus* enhanced growth of *Oryza sativa* seedlings under Cd stress, due to enhanced activity of antioxidant enzymes and reduced EL. Current research also showed that *B. thuringiensis* IAGS 199, alone or in combination with Put, alleviated Cd toxicity through enhanced activity of antioxidative enzymes and reduction of EL. 

The results of the current study demonstrated that Cd stress enhanced the amount of corresponding chelating biochemicals including protein and non-protein bound thiols as well as total thiols. The sulfhydryl groups of these chelating biochemicals successfully make compounds with metals causing a reduction in bioavailability and uptake of metals by plants [83]. Heavy metal transporting ATPases including Zip and Nramp enable the transport of phytochelatins bounded metal complexes in plant vacuoles [84]. Transformation, immobilization and mobilization of metal ions decrease metal uptake and toxicity in PGPB assisted plants by bio-accumulating phenomenon comprising sequestration, biosorption and bioexclusion, complexation and exclusion, with metal-binding proteins [85]. The amino acids and organic acids present in root exudates engage ligands including MTs and phytochelatins in the process of chelation and precipitation to influence bioavailability, solubilization and mobilization of metal ions in growing media [86,87]. The results of current research are in agreement with the findings of Aly and Mohamed [88] in *Zea mays* plants growing under copper regimes. Nagalakshmi and Prasad [89] also observed that higher Cu contents induced modulation in glutathione synthesis resulting in binding and sequester of Cu in stressed *Scenedesmus bijugatus*. Awasthi et al. [90] reported that thiol metabolism comprising phytochelatins, glutathione, protein and non-protein thiols (NBTs) make metal compounds, reduce metal uptake and sequester it in plant vacuoles resulting in the mitigation of metal toxicity in plants. Additionally, NBT persuades antioxidative activities in plants. Cysteine synthesized during sulphur assimilation of thiol metabolism improves biosynthesis of glutathione and phytochelatins. Awasthi et al. [91] reported metal complexation in tissues resulted in a higher level of phytochelatins, glutathione, and NBTs in *P*. *putida* inoculated rice plants under As stress. Hassan and Bano [92] observed that exogenous Put enhanced uptake and bioaccumulation of Mg, K and Ca in plants subjected to salinity stress. The improved uptake of essential nutrients may have enhanced the synthesis of thiols and successively increased biosynthesis of metal chelating biochemical in microbe-assisted plants.

## 4. Materials and Methods 

### 4.1. Procurement of Capsicum annum L. Germplasm and Growth Conditions

The experiment was conducted in the Department of Botany, University of Narowal, Narowal with temperature 23 ± 4 °C, humidity: 70–76% and light: 500–550 mmol m^−2^ s^−1^ light. Seeds of the commonly cultivated *Capsicum annuum var. fasciculatum* were obtained from Punjab Seed Corporation Pakistan and were used as per requirement of the study. The obtained *C. annum* seeds with initial moisture contents of 9.8% were sterilized through submerging in sodium hypochlorite solution (0.5%) for 3 min followed by washing thoroughly with distilled water [93]. These seeds were primed with 1 mM Put at 25 °C for 15 h under dark conditions. Seeds were dried by placing over blotting paper at 25 °C for 24 h before sowing in pots. 

The soil used for the present study was obtained from Botanical Garden, University of the Narowal. Soil was autoclaved at 121 °C and 1.5 bars. Soil contents were measured before sterilization. Soil was unified by mixing it thoroughly and for pot experiment contained 0.35 g Potassium, 0.06 mg cadmium, 0.35 mg chromium, 2.7 g organic matter and 1.76 g nitrogen. Subsequently, soil was contaminated with Cd (50 mg kg^−1^) and 2 kg soil was filled in polystyrene pots. For this purpose, cadmium chloride (CdCl_2_) was thoroughly mixed in the soil. The Cd amended and non-spiked soil samples were placed in the shade for 15 days. Pots were placed under greenhouse conditions. Five seeds were sown in each pot. Soil samples were filled in plastic pots (6′′ × 5′′). Pots were provided 50 mL full strength Hogland’s solution alone or contaminated with Cd during each alternative day [94]. Pots that received full-strength Hoagland’s solution were regarded as control. Plants were watered on alternate days. Distilled sterilizer water was used for watering plants. After one month of sowing, seedlings were harvested carefully and were submerged in 20 mM EDTA for 15 min to eradicate adsorbed metal ions. Plant samples were frozen by applying liquid-N and stored at −80 °C for the upcoming physiochemical examination.

### 4.2. Procurement and Characteristics of Bacterial Agent

*B. thuringiensis* IAGS 199 was obtained from the bacterial conservatory, University of the Punjab, Lahore. Indole acetic acid synthesis capability of microbe was evaluated with the help of the method described by Glickmann and Dessaux [95]. For this purpose*, B. thuringiensis* IAGS 199 was grown in LB medium having tryptophan (500 mg) for 1 d. Afterwards, culture was centrifuged and 1 mL supernatant was mixed with 50 mL orthophosphoric acid (10 mM) and 2 mL of Salkowski’s reagent for 0.5 h. The absorbance value was determined at 530 nm and was calibrated with a known standard value of IAA. As far as IAA production capability of microbe is concerned, *B. thuringiensis* IAGS 199 was capable to synthesize IAA (13 µg mL^−1^).

Cadmium tolerance was examined by inoculating 10 mL of *B. thuringiensis* IAGS 199 inoculum on LB agar plates supplemented with 0, 50, 75, 100 mg kg^−1^ Cd at 25 °C for 4 d. The plates without Cd showed 5 mm bacterial colony. Bacterial growth was not observed above 75 mg kg^−1^ Cd.

A pilot experiment was also conducted to evaluate the effectiveness of seed priming with different concentrations of Put (0.25, 0.5, 1, 1.5 mM Put) in the alleviation of 50 mg kg^−1^ Cd concentration in *C. annum* seedlings. The survival percentage of *C. annum* seedlings increased significantly in *C. annum* seedlings treated with 1 mM Put. The survival percentage of *C. annum* seedlings treated with 0.25, 0.5, 1, 1.5 mM Put-treated seedlings were 65%, 58%, 84%, 72%, respectively, under 50 mg kg^−1^ Cd concentration.

### 4.3. Effect of B. thuringiensis IAGS 199 on Concentration of Water Soluble Cd in Solution 

Method of Chen [96] was adopted to analyze the immobility characterization of water-soluble Cd in *B. thuringiensis* IAGS 199 solution besides the concentration of water-soluble Cd and Put concentration in the solution [96]. *B. thuringiensis* IAGS 199 was inoculated in culture flasks containing 1.0 mg L^−1^ Cd^2+^ supplemented LB media. The un-inoculated 1.0 mg L^−1^ Cd^2+^ supplemented LB media was regarded as control. Treated flasks were placed at 28 °C for 0, 1, 3, 5 and 7 d. Afterwards, *B. thuringiensis* IAGS 199 growth from culture solution was measured. The pH of the *B. thuringiensis* IAGS 199 culture solution was also observed by using pH meter. The *B. thuringiensis* IAGS 199 culture solution was subjected to centrifugation for 5 min at 12,000 rpm. The amount of Cd present in the supernatant was measured by using inductively coupled plasma-optical emission spectrometer. 

### 4.4. Assessment of Put in the Culture Solution

The method adopted by Ebeed et al. [97] with a slight amendment was employed for Put estimation. A 4-mL sample from all aforementioned flasks was subjected to centrifugation for 10 min at 10,000 rpm at 4 °C. *B. thuringiensis* IAGS 199 cells were removed and the supernatant was passed through a 0.22 μm filter paper. The 2 mL filtrate was mixed with 2 mL of cold perchloric acid (10% *v*/*v*) before keeping at 4 °C for 60 min. The aliquot of the supernatant was vortexed with 15 mL of benzoyl chloride along with 2 mL of 2 mol L^−1^ NaOH and placed at 38 °C for 0.5 h. To stop the reaction, 4 mL saturated NaCl solution was added. Subsequently, the benzoyl Put was removed by using 3 mL cold diethyl ether. Then, 1.5 mL of the ether phase was evaporated to dryness after which 1 mL methanol was added. The mixture was vortexed and passed through a 0.45 μm porous membrane filter (Millipore). Put concentration from 2 μL filtrate was determined through HPLC (ACQUITY UPLC H-Class Core System; Waters: USA) supplemented with the column (ACQUITY UPLC HSS T3 (2.1 by 100 mm) 1.8 μm-pore-size). The UV-2487 detector, adjusted at 230 nm was used as a detection device. The acetonitrile/water (44:56, *v*/*v*) solution passed through a 0.22 μm pore size membrane filter was employed as a mobile phase which was supplied at 0.45 mL min^−1^ flow rate. The peak of Put was recognized and measured by comparing with known HPLC values of Put standard. The Put detection limit was 0.1 μg mL ^−1^.

### 4.5. Experimental Treatments

The treatments used for the present study are as follows:

1. Control (C): Un-inoculated soil without Cd contamination
2. Cadmium contaminated (Cd): Un-inoculated soil with 50 mg kg^−1^ Cd
3. Put: Seeds primed with Put and grown in un-inoculated soil without Cd contamination
4. PGPB: *B. thuringiensis* IAGS 199 inoculated soil without Cd contamination
5. Cd + Put: Seeds primed with Put and grown in un-inoculated soil having 50 mg kg^−1^ Cd contamination
6. Cd + PGPB: Inoculated soil with 50 mg kg^−1^ Cd contamination
7. Cd + Put + PGPB: Seeds primed with Put and grown in inoculated soil with 50 mg kg^−1^ Cd contamination.

Put (1 mM) primed seedlings of *C. annum* plants were grown in *B. thuringiensis* IAGS 199 inoculated soil contaminated with Cd (50 mg kg^−1^) for 30 d.

### 4.6. Analysis of Plant Samples

Harvested plant samples were separated into leaves, shoot and roots for different biochemical analyses. The growth parameters of treated plants for instance root fresh weight, shoot fresh weight; root dry weight and shoot dry weight were analyzed according to Anwaar et al. [98]. Fresh biomass of plant samples was measured followed by drying these samples till constant weight in an oven at 70 °C for 48 h to analyze dry biomass.

### 4.7. Estimation of Chlorophyll Contents

For assessment of total chlorophyll contents (Chl a and Chl b), 100 mg fresh weight of foliage sample was grounding along with 8 mL of acetone 80% (*v*/*v*) in a pre-chilled mortar. The mixture of extract was filtered and the volume adjusted to 10 mL by adding cold acetone. The colorimetric value of the supernatant was observed at 663.2, 646.8 nm regarded as the amount of Chl a, Chl b correspondingly [99,100].

### 4.8. Determination of Photosynthetic Rate and Gas-Exchange Parameters

The net photosynthesis rate (*A*)*,* transpiration rate (*E*) and intercellular CO_2_ concentration was determined early in the morning (at 9 am) in the leaves with the help of portable gas-exchange system (Li-COR Inc., Biosciences, Lincoln, NE, USA) according to the methodology of Holá et al. (2010).

### 4.9. Determination of Total Soluble Protein 

The magnitude of total soluble protein was assessed at 595 nm by using bovine serum albumin according to Bradford [101].

### 4.10. Assessment of Soluble Sugars

Phenol sulphuric acid method was employed for the evaluation of soluble sugars as described by Dubois et al. [102]. Homogenized 0.5 g plant sample was added in test tubes containing 80% ethanol (10 mL). This solution was heated over water bath at 80 °C for 60 min and mixture (0.5 mL) was transferred to other tubes. An equal volume of deionized water along with 1 mL of 18% phenol was mixed. Tubes were permitted to cool at 25 °C for 30 min after which absorbance was noted at 490 nm. The number of total sugars present in the sample was estimated according to the following equation:

$$\mathrm{Sugar}=\left(\frac{\mathrm{Sample}\;\mathrm{absorbance}\times \mathrm{dilution}\;\mathrm{factor}\times K\;\mathrm{value}}{\mathrm{weight}\;\mathrm{of}\;\mathrm{fresh}\;\mathrm{plant}\;\mathrm{tissue}}\right)$$

### 4.11. Determination of Electrolyte Leakage (EL)

The totally extended topmost leaves were randomly selected and cut into 0.5 cm sections. The leaf section submerged into glass tubes containing 7 mL sterilized water. Tubes containing these leaves section were retained over rotary shaker for 1 day at 25 °C. The readings for primary conductivity (ECi) of the leaf sections were calculated by autoclaving the section holding tubes for 30 min at 120 °C. Values for maximum conductivity (ECmax) from a solution containing leaves section were estimated at 25 °C for measuring of EL in line with the following formula described by Li et al. [103]:

$$\mathrm{EL}\;\left(\%\right)=\frac{\mathrm{ECi}}{\mathrm{ECmax}}\;\times 100$$

### 4.12. Evaluation of Antioxidant Enzymatic Activities

The antioxidative enzymes including SOD, POD and CAT were estimated with the help of MagNA Lyser and 1 mM ASC (Roche, Vilvoorde, Belgium). For evaluation of SOD activity, fresh leaves sample (100 mg) was homogenized along with solutions including 0.25% (*v*/*v*) Triton X-100, 10% (*w*/*v*) polyvinylpyrrolidone (PVP), 1 mM phenylmethylsulfonyl fluoride (PMSF), 1 mL of 50 mM potassium phosphate buffer at pH 7.0. Decrease in NBT (nitroblue tetrazolium) was measured at 560 nm to estimate SOD activity [104].

For the estimation of POD activity, leaf samples (1 g) were vortexed with 3 mL of KH_2_PO_4_ buffer 100 mM (pH = 7). The homogenate was subjected to centrifugation at 12,000 rpm for 20 min at 4 °C. The reaction mixture was arranged by overtaxing 100 mL supernatant with 50 mL of guaiacol solution having 3 mL of KH_2_PO_4_ buffer and 30 mL of H_2_O_2_. The absorbance of the mixture was estimated by using spectrophotometer at 436 nm according to Putter [105].

For evaluation of CAT activity, 1.0 g leaf sample was homogenized with 3 mL of 100 mM KH_2_PO_4_ buffer at pH = 7 followed by 20 min centrifugation at 12,000 rpm at 4 °C. The supernatant (70 mL) was homogenized with 1500 mL 50 mM KH_2_PO_4_ buffer and 930 mL of 15 mM. H_2_O_2_. The amount of H_2_O_2_ decomposed at 240 nm was estimated to assess CAT activity [106]. 

For determination of APX activity, 1 g leaf sample was mixed in solution comprehending 10 mM 4-(2-Hydroxyethyl)-1-piperazinepropanesulfonic acid, 25 mL of *N*-(2-Hydroxyethyl) piperazine-*N*′-(3-propanesulfonic acid), polyvinylpyrrolidone (2%), EDTA (0.2 mM) at neutral pH. Before centrifugation, filtration of the solution was carried out with the help of nylon mesh. The solution was centrifuged at 5 °C for 20 min. The supernatant (0.2 mL) obtained was mixed with ascorbic acid (0.25 mM), EDTA (0.1 mM), phosphate buffer (25 mM) at neutral pH. Then H_2_O_2_ (1 mM) was added and level of oxidation carried out by ascorbate was observed at 290 nm. Oxidation was again recorded after 1 min. The difference obtained in both the spectrometrically calculated values was then divided to molar co-efficient of ascorbate [107].

### 4.13. Determination Proline Contents 

For estimation of proline, 1.0 g of leaf tissues was homogenized in 10 mL (3%) sulfosalicylic acid and kept at 100 °C for 15 min. Afterwards, 4 mL ninhydrin and 4 mL glacial acetic acid were dissolved and reserved for 60 min at 90 °C. After cooling, 8 mL toluene was mixed and absorbance was observed at 520 nm [108].

### 4.14. Analysis of Cadmium Content

Plant samples were uprooted and washed by using distilled H_2_O. Plant samples were oven-dried for 48 h (hrs). Digestion of oven-dried plant samples was carried out in HNO_3_: HClO_4_. Cadmium content in digested plant samples was quantified with the help of atomic absorption spectrophotometer. The quantity of Cd content in digested plant samples was carried out by multiplication of dry weight with Cd content in plant tissues. 

For assessment of translocation factor (TF), Cd content in the shoot was divided into the root Cd content according to Mattina et al. [109]. Metal tolerance index (MTI) was measured by the following equation:

$$\mathrm{MTI}=\frac{\mathrm{DWTP}\;}{\mathrm{DWNP}}\times 100$$

where DWPS = dry weight of PGPB-treated plant, DWNP = dry weight of control seedlings.

### 4.15. Determination of Total Thiols

Sedlak and Lindsay [110] method was employed for the assessment of total thiols. For this, plant sample (0.5 g) was homogenized in ascorbate buffer (20 mM) prepared with the help of EDTA (20 mm). Afterwards, centrifugation was accomplished at 12,000× *g* for 20 min at 4 °C. The supernatant (0.5 mL) attained was mixed with Tris HCl (200 Mm) and 10 mM of 5,5-dithio-bis-[2-nitrobenzoic acid]. The mixture was permitted to stand for 20 min and absorbance was calculated at 412 nm. 

### 4.16. Determination of Non-Protein Thiols

Plant sample (0.5 g) was macerated in 5% sulphosalicylic acid (3 mL). Afterwards, the samples were centrifuged at 12,000× *g* for 20 min at 4 °C. The extract (100 µL) obtained was mixed with 0.1 M potassium phosphate buffer and 0.5 mL of 1 Mm 5,5-dithio-bis-[2-nitrobenzoic acid]. The mixture was then allowed to stand for 20 min and absorbance value was calculated at 412 nm [111].

### 4.17. Determination of Protein Bound Thiols

Protein-bound thiols were calculated by subtraction of non-protein thiol from total thiols.

### 4.18. Statistical Analysis

One-way analysis of variance (ANOVA) was conducted and seven treatment means were compared using Duncan’s multiple range test (DMRT) [112]. The differences were considered significant when *p*-value was at least ≤ 0.05. DSASTAT statistical package software was employed for statistical analysis.

## 5. Conclusions

The results of the current study demonstrate that the presence of Cd in the rhizospheric area has detrimental effects on germination, seedling growth and biomass production of *C. annum*. Nevertheless, an affirmative effect of seed priming with Put and application of *B. thuringiensis* IAGS 199 significantly reinstates the physiological and morphological growth parameters of plants. Higher Cd tolerance in Put and *B. thuringiensis* IAGS 199-treated seedlings was attributed to the increased biosynthesis of osmoregulators including proline in consort with improved total soluble proteins and soluble sugars. Additionally, the reduced level of EL and MDA in plant tissues also abridged the Cd-induced toxicity. Furthermore, Put and *B. thuringiensis* IAGS 199 reduced bioavailability and uptake of Cd through binding it within root tissues by synthesizing chelating compounds including protein and non-protein thiols. Consequently, the present study advocates the application of Put and *B. thuringiensis* IAGS 199 as phytostabilizers for the successful cultivation of *C. annum* under Cd contaminated conditions. At the moment, it would be of pronounced interest to evaluate the transcriptome based strategy(s) by which Put and *B. thuringiensis* IAGS 199 maintain the physiochemical activities of plants under Cd stress. 

## Figures and Tables

**Table 1:** Effect of Put and *B. thuringiensis* IAGS 199 on root fresh weight, shoot fresh weight, root dry weight and shoot dry weight of *C. annum* seedlings under Cd stress.

Treatments	Germination (%)	Root FW (g plant^−1^)	Shoot FW (g plant^−1^)	Root DW (g plant^−1^)	Shoot DW (g plant^−1^)
C	82 ± 5.35 bc	2.45 ± 0.65 cd	6.54 ± 1.26 cd	1.76 ± 0.23 c	2.27 ± 0.56 c
Cd	57 ± 3.56 d	1.34 ± 0.18 d	4.12 ± 1.03 e	1.25 ± 0.28 d	1.46 ± 0.37 d
Put	86 ± 6.24 b	3.16± 0.75 b	7.12 ± 1.78 bc	2.12 ± 0.78 bc	3.87 ± 1.14 b
PGPB	92 ± 7.82 a	4.82 ± 0.67 a	8.76 ± 1.98 a	3.08 ± 1.54 a	4.65 ± 1.56 a
Cd + Put	65 ± 3.47 cd	1.02 ± 0.19 de	4.87 ± 1.18 d	1.67 ± 0.68 cd	2.45 ± 0.82 cd
Cd + PGPB	74 ± 4.62 c	2.76 ± 0.78 c	6.76 ± 1.72 c	2.65 ± 0.87 b	3.51 ± 1.06 bc
Cd + Put + PGPB	86 ± 6.54 b	3.16 ± 0.47 b	7.86 ± 1.94 b	2.98 ± 0.91 ab	4.23 ± 1.02 ab

Different letters indicate significant difference among the treatments (*p* ≤ 0.05). C, control; Cd, 50 mg kg^−1^ Cd; Put, 1 mM Put; PGPB, *B. thuringiensis* IAGS 199.

**Table 2:** Effect of Put and *B. thuringiensis* IAGS 199 on Chl a, Chl a, total Chl content, soluble sugar and total soluble proteins of *C. annum* under Cd stress.

Treatments	Chla	Chlb	Total Chlorophyll	Soluble Sugars (mg g^−1^ DM)	Total Soluble Proteins (μg g^−1^)
C	0.75 ± 0.13 cd	0.56 ± 0.16 cd	1.31 ± 0.65 cd	5.76 ± 1.03 d	0.75 ± 0.16 d
Cd	0.56 ± 0.14 d	0.29 ± 0.12 d	0.85 ± 0.15 d	6.87 ± 1.34 c	0.45 ± 0.11 de
Put	1.22± 0.56 bc	1.04 ± 0.23 b	2.26 ± 0.45 bc	7.56 ± 1.56 b	1.02 ± 0.34 bc
PGPB	1.92 ± 0.17 a	1.56 ± 0.37 a	3.48 ± 0.89 a	8.65 ± 1.84 a	1.34 ± 0.54 ab
Cd + Put	0.76 ± 0.18 cd	0.57 ± 0.17 cd	1.33 ± 0.27 cd	6.56 ± 1.65 cd	0.85 ± 0.12 c
Cd + PGPB	0.92 ± 0.67 c	0.87 ± 0.14 c	1.79 ± 0.48 c	7.43 ± 1.87 bc	1.12 ± 0.52 b
Cd + Put + PGPB	1.32 ± 0.62 b	1.02 ± 0.78 bc	2.34 ± 0.67 b	8.39 ± 1.97 ab	1.45 ± 0.78 a

Different letters indicate significant difference among the treatments (*p* ≤ 0.05). C, control; Cd, 50 mg kg^−1^ Cd; Put, 1 mM Put; PGPB, *B. thuringiensis* IAGS 199.

**Table 3:** Effect of Put and *B. thuringiensis* IAGS 199 on Cd content in root and shoot, translocation factor (TF) and metal tolerance index (MTI) in *C. annum*.

Treatments	Cadmium Content			
	Root (mg kg^−1^)	Shoot (mg kg^−1^)	TF	MTI
C	ND	ND	ND	-
Cd	12671 ± 78 a	9650 ± 36 a	0.76 ± 0.02 a	40.41 ± 4.42 e
Put	0.21 ± 0.02 de	0.10 ± 0.02 e	0.47 ± 0.11 ab	134.84 ± 8.58 bc
PGPB	0.38 ± 0.05 d	0.13 ± 0.02 e	0.34 ± 0.03 b	162.02 ± 11.56 a
Cd + Put	7435 ± 0.05 b	576 ± 24 b	0.07 ± 0.02 c	85.36 ± 3.75 d
Cd + PGPB	6185 ± 43 c	465 ± 17 c	0.07 ± 0.01 c	122.29 ± 6.21 c
Cd + Put + PGPB	7237 ± 24 bc	206 ± 13 d	0.02 ± 0.01 d	147.36 ± 7.34 b

Different letters indicate significant difference among the treatments (*p* ≤ 0.05). C, control; Cd, 50 mg kg^−1^ Cd; Put, 1 mM Put; PGPB, *B. thuringiensis* IAGS 199.

**Table 4:** Effect of Put and *B. thuringiensis* IAGS 199 on metal chelating compounds of *C. annum* under Cd stress.

Treatments	Total Thiols (mmol g^−1^ FW)	Non-Protein Bound Thiols (mmol g^−1^ FW)	Protein Bound Thiols
C	0.24± 0.012 d	0.13 ± 0.014 d	0.11 ± 0.015 cd
Cd	0.37 ± 0.016 cd	0.18 ± 0.012 cd	0.19 ± 0.014 d
Put	0.29 ± 0.0015 cd	0.17 ± 0.010 cd	0.12 ± 0.0072 c
PGPB	0.46 ± 0.0017 bc	0.21 ± 0.013 bc	0.25 ± 0.0067 b
Cd + Put	0.39± 0.0045 c	0.19 ± 0.011 c	0.18 ± 0.0054 bc
Cd + PGPB	0.67 ± 0.0012 b	0.25 ± 0.014 a	0.42 ± 0.0061 ab
Cd + Put + PGPB	0.91 ± 0.0017 a	0.23 ± 0.015 b	0.68 ± 0.008 a

Different letters indicate significant difference among the treatments (*p* ≤ 0.05). C, control; Cd, 50 mg kg^−1^ Cd; Put, 1 mM Put; PGPB, *B. thuringiensis* IAGS 199.
